# A Southeast Asian expert consensus on the management of major depressive disorder with suicidal behavior in adults under 65 years of age

**DOI:** 10.1186/s12888-022-04140-6

**Published:** 2022-07-21

**Authors:** Kok Yoon Chee, Nalini Muhdi, Nor Hayati Ali, Nurmiati Amir, Carmina Bernardo, Lai Fong Chan, Roger Ho, Pichai Ittasakul, Patanon Kwansanit, Melissa Paulita Mariano, Yee Ming Mok, Duy Tam Tran, Thi Bich Huyen Trinh

**Affiliations:** 1 NEURON, Department of Psychiatry & Mental Health, Kuala Lumpur Hospital, Kuala Lumpur, Malaysia; 2 Department of Psychiatry, Dr Soetomo General Hospital; Faculty of Medicine, Universitas Airlangga, Surabaya, Indonesia; 3 Department of Psychiatry & Mental Health, Selayang Hospital, Selayang, Selangor Malaysia; 4 Department of Psychiatry, Ciptomangunkusumo Hospital; Faculty of Medicine, Universitas Indonesia, Jakarta, Indonesia; 5 Mood and Anxiety Resource and Referral Center, Professional Services, Department of Neuroscience, Makati Medical Center, Makati City, Philippines; 6 Department of Psychiatry, Faculty of Medicine, University Kebangsaan Malaysia, Kuala Lumpur, Malaysia; 7 Department of Psychological Medicine, National University Hospital, Singapore, Singapore; 8 Department of Psychiatry, Faculty of Medicine, Ramathibodi Hospital, Mahidol University, Bangkok, Thailand; 9 Somdejchaopraya Psychiatry Institute, Bangkok, Thailand; 10 Department of Psychiatry, University of the East Ramon Magsaysay Memorial Medical Center, Quezon City, Philippines; 11 Institute of Mental Health, Singapore, Singapore; 12 Ho Chi Minh Psychiatric Hospital, Ho Chi Minh, Vietnam; 13 Bach Mai Hospital, Hanoi, Vietnam

**Keywords:** Depression, Suicide, Treatment, Southeast Asia, Consensus

## Abstract

**Background:**

The high prevalence of suicidal behavior among individuals with major depressive disorder (MDD) in Southeast Asia (SEA) underscores the need for optimized management to address depressive symptoms, reduce suicide risk and prevent suicide in these individuals. Given the lack of clear guideline recommendations for assessing and managing these patients, regional consensus-based recommendations which take into account diverse local contexts across SEA may provide useful guidance for clinical practice.

**Methods:**

A narrative literature review and pre-meeting survey were conducted prior to the consensus meeting of an SEA expert panel comprising 13 psychiatrists with clinical experience in managing patients with MDD with suicidal behavior. Utilizing the RAND/UCLA Appropriateness Method, the expert panel developed consensus-based recommendations on the assessment and treatment of adult patients with MDD with suicidal behavior under 65 years.

**Results:**

Screening of adult patients under 65 years with MDD for suicide risk using both a validated assessment tool and clinical interview is recommended. An improved suicide risk stratification – incorporating both severity and temporality, or using a prevention-focused risk formulation – should be considered. For a patient with an MDD episode with *low risk of suicide*, use of antidepressant monotherapy, and psychotherapy in combination with pharmacological treatment are both recommended approaches. For a patient with an MDD episode with *high risk of suicide*, or *imminent risk of suicide requiring rapid clinical response*, or for a patient who had received adequate AD but *still reported suicidal behavior*, recommended treatment strategies include antidepressant augmentation, combination use of psychotherapy or electroconvulsive therapy with pharmacological treatment, and inpatient care. Suicide-specific psychosocial interventions are important for suicide prevention and should also be part of the management of patients with MDD with suicidal behavior.

**Conclusions:**

There are still unmet needs in the assessment of suicide risk and availability of treatment options that can deliver rapid response in patients with MDD with suicidal behavior. These consensus recommendations on the management of adult patients with MDD with suicidal behavior under 65 years may serve as a useful guidance in diverse clinical practices across the SEA region. Clinical judgment based on careful consideration of individual circumstances of each patient remains key to determining the most appropriate treatment option.

**Supplementary Information:**

The online version contains supplementary material available at 10.1186/s12888-022-04140-6.

## Background

Suicide, a leading cause of death worldwide, is a major public health issue – approximately 800,000 people die by suicide every year [1]. Suicide rates are higher in younger adults [1], resulting in substantial years of life lost and associated socioeconomic burden. In 2016, the global age-standardized suicide rate was 10.5 (per 100,000 population); among the Southeast Asian (SEA) nations, Thailand has the highest age-standardized suicide rate in 2016 (12.9), followed by Singapore (7.9), Vietnam (7.0), Malaysia (6.2), Indonesia (3.7) and the Philippines (3.7) [1].

Suicidal behavior – which includes suicidal ideation, suicidal plan and suicidal acts (such as suicide attempt and suicide itself) [2] – is associated with several mental disorders, including major depressive disorder (MDD) [3]. Studies have shown that depressive symptoms, such as feeling of hopelessness or worthlessness, are risk factors for suicidal behavior [3, 4]. Indeed, individuals with MDD have a 20-fold greater risk of suicide than the general population [5]; approximately, 55% of individuals with MDD experience suicidal ideation [6]. A recent meta-analysis of observational surveys reported that suicide attempt is common in individuals with MDD, with a pooled lifetime prevalence of 31% [7]. In SEA, the reported prevalence of MDD with suicidal behavior (MDSB) ranges between 12.3 and 43.6% [8–13].

The high prevalence of suicidal behavior among individuals with MDD thus underscores the need for optimized management to manage depressive symptoms, reduce suicide risk, and prevent suicide in these individuals. However, there are several barriers which render the assessment and management of suicide risk in individuals with MDD complicated. Patients with MDD may be reluctant to speak about suicidal behavior due to cultural or religious beliefs and public stigma [14–17], making accurate assessment of suicide risk difficult. While international and local guidelines for the management of MDD generally state the necessity of a thorough and ongoing evaluation of suicide risk in all patients with MDD [18–22], there is a lack of clear recommendations on how to assess and manage patients with MDSB, and a lack of guidance for an integrated biopsychosocial approach to prevent suicide in these patients. The use of suicide risk assessment scales provides a standardized measure to support clinical interviews [23], but information collected is subjective. There is also a lack of treatment recommendations for patients with MDD at high risk of suicide [24], highlighting a need for treatment options that can rapidly reduce suicidal behavior. These barriers are further compounded by time constraints faced by clinicians [23] and limited access to care and treatment in some parts of Asia [14].

Given the significant impact of suicide in SEA and the lack of clear guideline recommendations for assessing and managing patients with MDSB, a regional consensus on the topic will be useful in providing guidance for clinical practice. Our manuscript aims to provide consensus-based recommendations on the assessment and management of patients with MDSB, taking into consideration the diverse local contexts across SEA.

## Methods

The consensus meeting was convened virtually in January 2021, bringing together an expert panel of 13 national psychiatrists from across SEA – including Indonesia, Malaysia, Philippines, Singapore, Thailand and Vietnam – to discuss and develop an SEA consensus on the management of patients with MDSB. All panelists have relevant knowledge and clinical experience in the management of patients with MDSB.

**Fig. 1:**
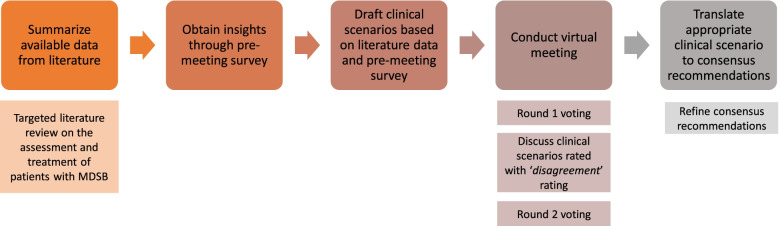
Consensus process. Abbreviations: MDSB, major depressive disorder with suicidal behavior

Prior to the consensus meeting, a targeted, narrative literature review on the assessment and treatment of adult patients with MDSB, as well as relevant guidelines, was conducted and the available information was summarized. Search terms included suicide, suicidality, suicidal ideation, suicidal behavior, suicide attempt, suicide risk, depression, major depressive disorder, guidelines, consensus, Singapore, Malaysia, Indonesia, Philippines, Thailand, and combinations of mentioned terms. Search criteria were limited to adult population and PubMed publications from 2010 to mid-November 2020 in English language. A separate search beyond PubMed was also conducted for existing guidelines. A pre-meeting survey was distributed to the expert panel to obtain insights on current practice patterns and unmet needs in their respective countries. Based on the available information from the literature review and insights from the pre-meeting survey, a set of clinical scenarios for the assessment and treatment of MDSB in adult patients under 65 years was drafted for the consensus development (Fig. 1). The narrative literature review was conducted by a medical writer from an independent medical communications agency (In Vivo Communications [Asia] Pte Ltd). Following discussions with the lead author (chairperson on the consensus meeting), a pre-meeting survey and clinical scenarios were also developed.

To obtain consensus, RAND/UCLA Appropriateness Method was employed – this method incorporates evidence from the literature as well as expert clinical opinions [25], and has been used for the development of guidelines in psychiatric settings [26]. During the meeting, the expert panel independently and confidentially rated the appropriateness of each clinical scenario or statement based on a 1 to 9 Likert scale in two voting rounds. Discrepancies in the ratings were identified following the first round of voting. ‘*Disagreement*’ was indicated when one-third or more panelists rated a statement/scenario in the lowest 3 points of the appropriateness scale (score 1, 2 or 3) and one-third or more panelists rated the same statement/scenario in the highest 3 points of the appropriateness scale (score of 7, 8 or 9). In the absence of ‘*disagreement*’, a rating with a median score of 7–9 was considered ‘*appropriate*’; median score of 4–6 was ‘*equivocal*’; and median score of 1–3 was ‘*not appropriate*’. Following the first round of voting, statements/scenarios indicated with ‘*disagreement*’ were discussed among the panel members before proceeding to the second round of voting. The results of the second round of voting were collated and were classified based on the pre-defined appropriateness rating. After the consensus meeting, ‘*appropriate*’ statements/scenarios were translated into consensus recommendations, which were further refined by the expert panel.

## Results

### Screening and assessment of suicide risk in patients with MDD

The expert panel recommends that all adult patients under the age of 65 years with MDD presenting to emergency departments or psychiatric hospitals should be screened for suicide risk.

Combining a validated assessment tool and clinical interview is recommended when assessing suicide risk in adult patients under 65 years with MDD. The expert panel considered the use of assessment tools alone (one or more tools) as ‘*inappropriate*’, while employing clinical interview alone for the assessment of suicide risk was rated as ‘*equivocal*’.

### Stratification of suicide risk in patients with MDSB

The expert panel recommends that the management of patients with MDSB should involve structured suicide risk assessment, risk stratification and the development of an individualized safety plan.

The expert panel was ‘*equivocal*’ about the sufficiency of traditional stratification based on severity (low risk vs. high risk) to formulate an individualized safety plan for patients with MDSB. The following stratification strategy should be considered:

- Stratification based on severity (low vs. intermediate vs. high risk) and temporality (acute vs. chronic)
- Prevention-focused risk formulation, taking into account 1) a patient’s risk relative to a specified population; 2) a patient’s risk over time; 3) resources available to the patient during crisis; and 4) foreseeable changes that may exacerbate suicide risk.

### Treatments for patients with MDD with suicidal behavior

Several factors should be considered while determining the nature and intensity of treatment for adult patients with MDSB:

- Severity of suicide risk
- Past and family history of suicidal attempt
- Presence of suicidal thought or ideation
- Access to suicide means
- Substance abuse
- Presence of social support network
- Treatment availability and accessibility
- Psychiatric comorbidities.

### Pharmacological and non-pharmacological treatment strategies

For the treatment of patients with MDSB, seventeen clinical scenarios were rated ‘*appropriate*’; 10 clinical scenarios were rated as ‘*equivocal*’; ‘*disagreement*’ was indicated in seven clinical scenarios, while the remaining was considered ‘*inappropriate*’ by the expert panel (Table 1).

**Table 1:** Consensus on the treatment of adult patients under 65 years with MDSB

Indication	Ratings
**An adult patient under 65 years with no significant organic or psychiatric history, has an MDD episode with a low risk of suicide. To reduce the risk of suicide**	
**1.** use one AD	**Appropriate**
**2.** use two ADs from the same class	**Not appropriate**
**3.** use two ADs from different classes	**Disagreement**
**4.** add lithium	**Not appropriate**
**5.** add buspirone	**Not appropriate**
**6.** add ketamine	**Not appropriate**
**7.** add intranasal esketamine^a^	**Not appropriate**
**8.** add benzodiazepines	**Equivocal**
**9.** add conventional antipsychotics	**Not appropriate**
**10.** add atypical antipsychotics	**Equivocal**
**11.** add thyroid supplements	**Not appropriate**
**12.** use ECT monotherapy	**Not appropriate**
**13.** use ECT in combination with AD(s) with or without adjuvants	**Not appropriate**
**14.** use rTMS monotherapy	**Not appropriate**
**15.** use rTMS in combination with AD(s) with or without adjuvants	**Not appropriate**
**16.** use psychotherapy alone	**Disagreement**
**17.** use psychotherapy in combination with AD(s) with or without adjuvants	**Appropriate**
**18.** admit for inpatient care	**Not appropriate**
**An adult patient under 65 years with no significant organic or psychiatric history, has an MDD episode with a high risk of suicide. To reduce the risk of suicide**	
**19.** use one AD	**Not appropriate**
**20.** use two ADs from the same class	**Not appropriate**
**21.** use two ADs from different classes	**Disagreement**
**22.** add lithium	**Equivocal**
**23.** add buspirone	**Not appropriate**
**24.** add ketamine	**Disagreement**
**25.** add intranasal esketamine^a^	**Appropriate**
**26.** add benzodiazepines	**Equivocal**
**27.** add conventional antipsychotics	**Not appropriate**
**28.** add atypical antipsychotics	**Appropriate**
**29.** add thyroid supplements	**Not appropriate**
**30.** use ECT monotherapy	**Not appropriate**
**31.** use ECT in combination with AD(s) with or without adjuvants	**Appropriate**
**32.** use rTMS monotherapy	**Not appropriate**
**33.** use rTMS in combination with AD(s) with or without adjuvants	**Equivocal**
**34.** use psychotherapy alone	**Not appropriate**
**35.** use psychotherapy in combination with AD(s) with or without adjuvants	**Appropriate**
**36.** admit for inpatient care	**Appropriate**
**If an adult patient under 65 years with no significant organic or psychiatric history, has an MDD episode with an imminent risk of suicide requiring rapid clinical response**	
**37.** use one AD	**Not appropriate**
**38.** use two ADs from the same class	**Not appropriate**
**39.** use two ADs from different classes	**Disagreement**
**40.** add lithium	**Not appropriate**
**41.** add buspirone	**Not appropriate**
**42.** add ketamine	**Disagreement**
**43.** add intranasal esketamine^a^	**Appropriate**
**44.** add benzodiazepines	**Equivocal**
**45.** add conventional antipsychotics	**Not appropriate**
**46.** add atypical antipsychotics	**Appropriate**
**47.** add thyroid supplements	**Not appropriate**
**48.** use ECT monotherapy	**Not appropriate**
**49.** use ECT in combination with AD(s) with or without adjuvants	**Appropriate**
**50.** use rTMS monotherapy	**Not appropriate**
**51.** use rTMS in combination with AD(s) with or without adjuvants	**Equivocal**
**52.** use psychotherapy alone	**Not appropriate**
**53.** use psychotherapy in combination with AD(s) with or without adjuvants	**Appropriate**
**54.** admit for inpatient care	**Appropriate**
**An adult patient under 65 years with no significant organic or psychiatric history, had an MDD episode with suicidal behavior. If the patient had received AD for an adequate dose, duration and adherence, but still reported suicidal behavior**	
**55.** add lithium	**Equivocal**
**56.** add buspirone	**Not appropriate**
**57.** add ketamine	**Disagreement**
**58.** add intranasal esketamine^a^	**Appropriate**
**59.** add sodium valproate	**Equivocal**
**60.** add atypical antipsychotics	**Appropriate**
**61.** add thyroid supplements	**Not appropriate**
**62.** use ECT monotherapy	**Not appropriate**
**63.** use ECT in combination with AD(s) with or without adjuvants	**Appropriate**
**64.** use rTMS monotherapy	**Not appropriate**
**65.** use rTMS in combination with AD(s) with or without adjuvants	**Equivocal**
**66.** use psychotherapy alone	**Not appropriate**
**67.** use psychotherapy in combination with AD(s) with or without adjuvants	**Appropriate**
**68.** admit for inpatient care	**Appropriate**

^a^Use in SEA will depend on local approved indications

*Abbreviations*: *AD* antidepressant, *ECT* electroconvulsive therapy, *MDD* major depressive disorder, *MDSB* MDD with suicidal behavior, *rTMS* repetitive transcranial magnetic stimulation

For a patient with an MDD episode with **low risk of suicide**, the following treatment strategy is recommended:

- use of one antidepressant (AD) as monotherapy
- use of psychotherapy in combination with AD(s) with or without adjuvants.

For a patient with an MDD episode with **high risk of suicide,** or **imminent risk of suicide requiring rapid clinical response**, or for a patient with MDSB who **has received an AD** with adequate dose, duration and adherence, but **still reported suicidal behavior**, the following treatment strategy is recommended:

- addition of intranasal esketamine (use will depend on local approved indications)
- addition of atypical antipsychotics
- use of electroconvulsive therapy (ECT) in combination with AD(s) with or without adjuvants
- use of psychotherapy in combination with AD(s) with or without adjuvants
- admit for inpatient care.

### Indications for inpatient care

Inpatient care is recommended in patients with MDD with:

- Suicidal plan or act
- High risk of suicide
- History of suicidal attempt
- Psychiatric comorbidities
- Absence of support network
- Substance abuse.

The ‘presence of suicidal ideation’ and ‘long history of mental illness’ as criteria for inpatient care were rated as ‘*equivocal*’ by the expert panel.

### Psychosocial intervention

Psychosocial interventions are recommended for patients with MDSB (Table 2) in combination with pharmacological therapy. However, the expert panel was ‘*equivocal*’ regarding the use of no-suicide contract as a brief psychosocial intervention and the use of mentalization as a long-term psychosocial intervention.

**Table 2:** Consensus on psychosocial interventions for patients with MDSB

Brief psychosocial intervention	Long-term psychosocial intervention
Caring contacts	Cognitive behavioral therapy
Safety planning intervention	Interpersonal psychotherapy
Crisis response planning	Dialectic behavioral therapy
Volitional help sheet	Collaborative assessment and suicidality management
Attempted Suicide Short Intervention program	Acceptance and commitment therapy

*Abbreviations*: *MDSB* MDD with suicidal behavior

## Discussion

Given the high prevalence of suicidal behavior among patients with MDD, there is a significant need to optimize the management of these patients to manage depressive symptoms, reduce suicide risk and prevent suicide. Mental health professionals are expected to recognize and foresee the possibility of a patient engaging in suicidal behavior, and therefore must 1) conduct a thorough and structured suicide risk assessment and 2) formulate suicide risk management to guide treatment planning [27].

### Risk assessment

Assessment of suicide risk begins by engaging and having empathetic conversations with the patients and the persons accompanying them to build a therapeutic alliance. Based on the initial conversations and observation, a thorough suicide risk screening and assessment should be carried out to: 1) identify risk and protective factors, 2) ask specifically about suicide (inquire and ascertain presence of thoughts/ideation of self-harm and history of suicidal behavior; assess suicidal thought intensity and severity; ascertain access to suicide method/plan), 3) determine suicide risk level, and 4) formulate safety and treatment plan.

Several tools have been developed to assist with the assessment of suicide risk; commonly used tools include Mini-International Neuropsychiatric Interview (MINI) suicidality module, Beck Scale for Suicide Ideation and the Columbia-Suicide Severity Rating Scale [12, 23, 28]. Clinical guidelines do not generally recommend a specific assessment tool and note that these tools should not be used alone [19, 22] as each tool has different diagnostic accuracy for suicidal behavior [29] and there is no available evidence to support the use of suicide risk assessment tools in predicting suicidal behavior. The expert panel believe that clinicians should use a validated assessment tool in combination with a detailed clinical interview when assessing suicide risk in patients with MDD – this will increase the chance of capturing vital information to achieve a more accurate risk assessment. However, this recommended approach may not always be practical. In a high-volume/non-specialist mental health setting (i.e., primary care or emergency department), a comprehensive clinical interview may not be feasible, especially if patients do not present with overt suicidal behavior as the main clinical problem. In this case, a simple validated assessment tool, such as the PHQ-9, Ask Suicide-Screening Questions (ASQ; 5 questions; https://www.nimh.nih.gov/research/research-conducted-at-nimh/asq-toolkit-materials), or the Columbia protocol (6 questions; https://cssrs.columbia.edu/the-columbia-scale-c-ssrs/risk-identification/), may be used – a positive result will then necessitate a more in-depth clinical assessment of suicide risk to be conducted by either the primary frontline healthcare worker or a specialist mental healthcare worker, depending on local healthcare systems.

Likewise, relying upon clinical interview alone for the assessment of suicide risk may be unavoidable in situations where patients are too distraught, uncooperative or non-forthcoming. It is recommended that patients who have been identified with suicidal risk are referred to accessible specialist mental health services for a comprehensive assessment, including corroborative history from available sources, for further management.

Suicide risk level has traditionally been stratified based on severity (low, medium/intermediate and high) but this stratification has been increasingly considered inadequate to guide optimal clinical decision-making [27, 30]. The expert panel agreed that to accurately assess suicide risk and formulate an individualized safety plan for patients with MDSB, a better stratification strategy is required. A two-dimensional risk stratification based on both severity (refers to the seriousness of suicidal intent; categorized as low, intermediate, high) and temporality (refers to whether the risk is immediate [acute], or over a long term [chronic]) has been proposed to help clinicians identify situations where inpatient care is warranted while avoiding unnecessary inpatient admissions which may be counterproductive and harmful to protective factors that help mitigate long-term suicide risk (e.g., employment) (Table 3) [30].

**Table 3:** Essential features in the two-dimensional risk stratification model [30]

	Acute	Chronic
**High**	• SI with intent to die by suicide • Inability to maintain safety independent of external support/help	• Chronic SI, which may become acutely suicidal upon unpredictable situations (e.g., job or relationship loss) • Limited coping skills and limited ability to identify reasons for living • Increase or change in baseline mood, behavior and talk about suicide or dying
**Intermediate**	• SI to die by suicide • Ability to maintain safety, independent of external support/help	• Similar to high chronic risk but have a relatively balanced protective factors, coping skills, reasons for living and psychosocial stability
**Low**	• No current suicidal intent, and • No specific and current suicidal plan, and • No preparatory behaviors, and • Collective high confidence (including patient, care provider, family member) in the ability of the patient to independently maintain safety	• Little or no mental health, or • Significant mental illness but have abundance of coping strengths and resources • Have history of managing stressors without resorting to SI • Absence of history of self-directed violence; chronic SI; tendency toward highly impulsive, risky behavior; severe, persistent mental illness; marginal psychosocial functioning.

*Abbreviations*: *SI* suicidal ideation

A different model proposes doing away with categorical predictions and instead incorporates four distinct judgments to formulate an individualized intervention plan: (1) *a patient’s risk relative to a specified population* – determined from presence of family history of suicide, history of mental illness, history of suicidal behavior and history of abuse; (2) *a patient’s risk over time* – determined from a patient’s recent suicidal statements or behavior, current symptoms and degree of engagement with help resources; (3) *resources available to the patient during crisis*; and (4) *foreseeable changes that may exacerbate suicide risk* [31]. While further evaluation is required, the expert panel believe that such models can provide a more accurate assessment of suicide risk and appropriate safety plan for patients with MDSB.

### Treatment strategies

The treatment plan for managing patients with MDSB should address both depressive symptoms and suicidal components to effectively reduce the risk of suicide. While the severity of suicide risk is not the only factor when deciding treatment, it serves as a basis in formulating a treatment plan. Guideline-recommended treatment options for patients with MDSB include inpatient care, ADs and ECT [18, 32–35], clearly highlighting the limited treatment options that are available to effectively manage patients with MDSB. A careful re-evaluation of the diagnosis is necessary when patients with MDSB fail to benefit from routine treatments.

#### Adult patients under 65 years with MDD with low risk of suicide

AD is the first-line treatment for MDD – for *an adult patient with an MDD episode and low risk of suicide*, AD monotherapy is sufficient to manage the depressive symptoms. There is evidence suggesting that AD increases suicide risk in younger population below the age of 25 years, while no increased risk was seen for adults older than 25 years and AD had a protective effect against the development of suicidal ideation and behavior in adults 65 years and older [36]. The potential for increased risk of suicidality caused by AD in the younger population necessitates continuous close monitoring for side effects, but it should not deter treatment of MDD with AD as the risk of suicide associated with untreated depressive symptoms is high.

Combining two ADs from different classes may cause excessive side effects and affect compliance, and thus is not a suitable first-line strategy in this situation; however, this strategy can be trialed when the patient has failed 2 AD monotherapies from different classes [37]. The use of adjunctive treatment in this situation is generally considered not necessary by the expert panel; however, the addition of benzodiazepines or atypical antipsychotics may be considered. Adjunctive benzodiazepines may be useful in the short term to help preserve sleep quality and mitigate anxiety that is common in MDD [38]; benzodiazepines have a rapid onset of action and can therefore improve mood and decrease the risk of suicide before the effect of AD can set in. A Cochrane review found that combination of AD and benzodiazepines was indeed more effective than AD alone in improving depressive symptoms in the early phase [39]. However, there is a risk of misuse and dependence associated with the use of benzodiazepines [38]. Similarly, atypical antipsychotics may also help to hasten the alleviation of depressive symptoms [40] – some of the expert panel members however will only use it in combination with AD if the patient has severe MDD. It is important to note that there is a risk of misuse of or overdose with prescribed medications as a means of suicide – clinicians should take this into consideration when determining treatment options. Patients should be carefully monitored considering their individual risks and measures to minimize such risks (e.g., medications prescribed in restricted amount; medications ae kept by caregivers) should be implemented.

Psychotherapy is recommended for the treatment of mild-to-moderate MDD [18] and there is also some evidence of benefit for psychotherapy in reducing suicidal ideation and behavior, albeit modest [41–44]. Some of the expert panel members consider psychotherapy alone as an appropriate first-line treatment in patients with mild depressive symptoms and low risk for suicide, who are psychologically minded but are not keen on pharmacological treatment; however, others believe that it may be inappropriate as monotherapy and should be used in combination with ADs for an optimal outcome. As such, the combination of psychotherapy with ADs, with or without adjuvants, can be a useful strategy for patients with MDSB as they target different components. It is important to note that while the combination of psychotherapy and AD has been evaluated in the context of depressive symptoms [45], there is currently no empirical study evaluating this strategy for the reduction of suicide risk.

#### Adult patients under 65 years with MDD with high risk of suicide / imminent risk of suicide / those who had received AD but still have suicidal behavior

Adult patients with MDD with *high risk of suicide* or *imminent risk of suicide* require prompt intervention to prevent them from self-harm. While ADs are a recommended treatment option for patients with MDSB, the effect of ADs is delayed, often requiring weeks before optimal effect can be achieved [46]; their use is therefore limited in such critical situations. There was a disagreement among the expert panel members regarding the use of two ADs from different classes for this group of patients – there is no evidence to support the use of two ADs from different classes for achieving a more rapid response to reduce the risk of suicide. However, such an approach may be appropriate considering that some patients with MDD with a high risk or imminent risk of suicide may have severe depression. Asian patients with MDD with suicidal thoughts or suicidal acts have shown a poorer response to ADs and an increased burden of illness [8].

As an adjunctive to ADs, lithium has been shown to be effective in MDD [46]. Furthermore, long-term treatment with lithium has demonstrated a beneficial effect in suicide risk reduction [32, 47–49]. The addition of lithium for the treatment of patients with MDD with *imminent risk of suicide* is considered inappropriate as rapid clinical response cannot be achieved with lithium; however, the expert panel was ‘*equivocal*’ regarding its use in patients with MDD with *high risk of suicide* or in those who *had received AD but still have suicidal behavior*. Due to the potential benefit in reducing risk of suicide in the long term, augmentation of ADs with lithium can be considered in these patients, depending upon the urgency of the situation. Although there are concerns over dependence of benzodiazepines and potential sleep disorder following cessation of benzodiazepines, some of the expert panel members consider it to be useful in patients with MDD with *high risk or imminent risk of suicide* – as a short-term adjunctive agent, it helps to manage anxiety and insomnia; this may be particularly useful during the night when supervision is likely to be less stringent. The addition of atypical antipsychotics is considered appropriate for patients with MDD with *high risk, imminent risk of suicide,* or in those who *had received AD but still have suicidal behavior*. While the potential of direct anti-suicidal effects of atypical antipsychotics in mood disorders requires further research [50], atypical antipsychotics can achieve faster clinical response (within up to 1 week) in reducing depressive symptoms compared with AD monotherapy [51]. Atypical antipsychotics have thus been commonly used as an augmentation agent in the treatment of MDD, and would therefore indirectly reduce the risk of suicide in these patients through the reduction of depressive symptoms.

Sodium valproate has been shown to improve aggression, anxiety and impulsivity in patients with bipolar disorder [52]. There may be an element of impulsivity in patients with suicidal behavior, as impulsivity is thought to facilitate the transition from suicidal ideation to attempt [53]; as such, patients with *MDD who had received AD but still have suicidal behavior* may benefit from the addition of sodium valproate to their treatment regimen. However, sodium valproate does not have an immediate onset of action, and thus some of the expert panel members will instead consider other adjunctive agents such as benzodiazepines or ECT. Sodium valproate is also not a guideline-recommended adjunctive agent for depressive symptoms in Indonesia and Vietnam.

Studies have reported the benefit of intravenous (IV) ketamine in reducing suicidal risk in patients with MDD – a single dose of IV ketamine led to a reduction of suicide ideation within 90 minutes of administration [54–56], demonstrating that it can provide a rapid clinical response for patients with MDD with *high risk of suicide*, *imminent risk of suicide*, or *those still reporting suicidal behavior despite receiving AD*. However, there was a disagreement among the expert panel members on its use in these patient populations – while data suggests the benefit of ketamine in reducing suicide risk, it is neither approved by United States Food and Drug Administration (US FDA) nor in most of the countries in the SEA for this indication. In Indonesia, ketamine is only used as anesthesia and psychiatrists are not trained to administer ketamine while in the Philippines, it is commonly used as an induction agent for ECT. In Thailand, there is a ketamine clinic which helps patients with treatment-resistant depression and those with *high risk or imminent risk of suicide*, and it has been very useful in decreasing the risk of suicide within 24 hours in these patients. In Malaysia, a case series reported varied responses to serial intravenous ketamine among patients with treatment resistant mood disorder patients and suicidal behavior, in addition to comorbidities such as substance use and personality disorders [57].

Intranasal esketamine, the S-enantiomer of ketamine, has been approved for treatment-resistant depression in adults in conjunction with an oral AD [58–62]. Recently, intranasal esketamine demonstrated rapid reduction of depressive symptoms among patients with MDD who have active suicide ideation with intent in randomized, placebo-controlled phase 3 clinical studies. Patients with MDD with active suicidal ideation with intent who received intranasal esketamine and comprehensive standard of care achieved clinically meaningful and statistically significant improvement in depressive symptoms at 24 hours post-treatment compared with placebo and comprehensive standard of care; patients in both groups (esketamine/active and placebo/control) experienced rapid improvement in the severity of suicidality [63, 64], highlighting its potential role as a treatment option for patients with MDSB. Based on this data, the US FDA and the Malaysian National Pharmaceutical Regulatory Agency have approved its use to treat depressive symptoms in adults with MDD with acute suicidal ideation or behavior [56, 60]; the European Medicines Agency also approved its use as acute short-term treatment, in combination with an oral AD, for the rapid reduction of depressive symptoms, which constitute a psychiatric emergency [59]. As such, the expert panel considers intranasal esketamine as an appropriate treatment option for patients with MDD with *high risk of suicide, imminent risk of suicide* or in those who *have received AD but still have suicidal behavior* (its use in this patient population depends on local regulatory approval).

Available data suggest that ECT is effective in eliminating acute suicide risk in severely depressed patients [32] and it is recommended in patients with MDD or treatment-resistant depression who have suicidal ideation or high risk of suicide [18, 33–35]. However, relapse of depressive symptoms has been reported post-ECT [65]. Therefore, in patients with MDD with *high risk of suicide, imminent risk of suicide* or in those who *have received AD but still have suicidal behavior*, the use of ECT in combination with pharmacological therapy is recommended. There is some evidence suggesting anti-suicidal benefits of repetitive transcranial magnetic stimulation (rTMS) in depressive patients [66, 67], although specific data in patients with MDD *with high risk or imminent risk of suicide* is lacking. Furthermore, the treatment effect of rTMS may only be seen after several sessions over 2–3 weeks have been completed. The clinical experience in using rTMS in these patient populations in SEA is very limited, as it is not easily accessible or available. However, the use of rTMS in combination with pharmacological therapy may be considered in patients with MDD *who have received AD but still have suicidal behavior* – such a combination approach acts on different pathways and may thus provide benefit for patients.

#### Inpatient care

Hospitalization or inpatient care is a guideline-recommended treatment option for patients with high risk of suicide [18, 32]. Inpatient care should also be considered in patients with MDD with suicidal behavior, history of suicide attempt, psychiatric comorbidities, absence of support network, and substance abuse. The expert panel considered ‘suicidal ideation’ and ‘long history of mental illness’ as risk factors for suicide but these parameters do not directly reflect the severity of suicide risk and are therefore insufficient to definitively determine the need for inpatient care. Further clarification is required to qualify as criteria for inpatient care – for example, passive suicidal ideation or suicidal ideation without a specific plan has relatively lower risk than suicidal ideation with a specific plan or with access to lethal means; patients with suicidal ideation who have protective factors (e.g., support network, religion, safety plan, etc.) may have lower risk compared with those without or less protective factors. Similarly, chronic mental illness may carry different risk depending on the nature of the disease and symptoms (e.g., whether the symptoms have been resolved; if symptoms are episodic or treatment resistant, etc.). The expert panel highlighted the importance of considering the presence of other factors in conjunction with ‘suicidal ideation’ and ‘long history of mental illness’ to definitively assess the need for inpatient care.

#### Psychosocial intervention

While treatment targeting the underlying psychiatric disorder would help diminish the risk of suicide, suicide-specific interventions are also important for suicide prevention and should be part of the management of patients with MDSB. Evidence has demonstrated the benefit of various short- and long-term psychosocial interventions, including psychotherapy, in reducing suicide attempts [42–44, 68]. The Collaborative Assessment and Management of Suicidality approach, designed to manage individuals with suicidal ideation and behavior with a collaborative problem-solving technique, was shown to be promising in reducing suicide risk in adult population, suicidal outpatients and in inpatient setting [69–71]. There is also evidence suggesting the benefit of acceptance and commitment therapy in reducing suicidal ideation [72]. Mentalization as a long-term psychosocial intervention may provide some benefit when use in combination with pharmacological treatment and mentalization-based therapy has an evidence base for its use in patients with borderline personality disorder [73, 74]. However, the expert panel considered that there is insufficient evidence to recommend mentalization-based therapy to reduce suicide risk in patients with MDD. Additionally, mentalization-based therapy is not commonly practiced by trained therapists in SEA.

Short-term psychosocial interventions often focus on helping individuals to become aware of suicidal behavior, motivating them to seek help and engage in safety planning, as well as developing management strategies for future suicidal crises [17]. Brief intervention and contact and safety planning intervention or crisis response planning, have been shown to reduce the risk of suicide [75–77]. Volitional help sheet was found to be effective in reducing the repetition of self-harm following a suicide attempt [78] while the Attempted Suicide Short Intervention program reduced the number of suicide attempts [79]. The expert panel considered that the use of no-suicide contract does not guarantee suicide deterrence and may even obscure the actual suicide risk of the patient – patients may not disclose their suicidal intention due to concerns over disappointing their clinicians by breaking the contract. Furthermore, there is no evidence to suggest that the use of no-suicide contract is effective in preventing suicidal behavior and may even be counterproductive [80, 81]; clinical guidelines have thus cautioned its use [80].

We acknowledge that our recommendations are based on a consensus of an expert panel that is not multidisciplinary. Our consensus recommendations are guided by current evidence and are generally in line with the recommendations from available guidelines, but they also reflect the variations of practice in the SEA countries, including in healthcare system and resources, and the availability and accessibility of treatment options and services. Although not specifically discussed in this manuscript, we recognize the importance of cultural/religious beliefs and public stigma in affecting suicidality of an individual. Treatment costs, financial considerations and reimbursement systems, which may vary from one region to another, are also important factors that also influence treatment decisions for each patient.

It is evident that there are still unmet needs in this field regarding assessment of suicide risk and availability of treatment options that can deliver rapid response in patients with MDSB. Further development of tools that can incorporate both patient- and clinician-reported information, such as the Suicide Ideation and Behavior Assessment Tool [82], may improve the accuracy of suicide risk assessment in patients with MDSB. In recent years, there has been an increasing interest in the use of artificial intelligence (AI) and data science to improve suicide prediction and monitoring and identification of suicide risk [83, 84]. Accumulating observations thus far suggest a promising benefit of AI-based tools in helping identify individuals at risk of suicide [83, 84], suggesting its potential utility in supporting clinical management of patients with MDSB. Additionally, data on the benefit of ketamine and intranasal esketamine in these patients thus far are also promising – further evidence and approval of these new agents will provide more effective treatment options for patients with MDSB.

## Conclusion

In view of the lack of clear guideline recommendations in managing patients with MDSB, we have developed consensus recommendations for the management of these patients in the SEA region, considering diverse local contexts. We hope that this consensus can serve as a guide in clinical practice; it should not replace clinical judgement – when determining the most appropriate approach for the management of patients with MDSB, individual circumstances and benefit-risk balance should be considered carefully.

## Supplementary Information

**Additional file 1: **Supplementary information. Ratings of clinical scenario for achieving consensus.

## Data Availability

All data generated or analyzed during this study are included in this published article [and its supplementary information files].

## Abbreviations

AD: Antidepressant
ECT: Electroconvulsive therapy
IV: Intravenous
MDD: Major depressive disorder
MDSB: MDD with suicidal behavior
MINI: Mini-International Neuropsychiatric Interview
rTMS: Repetitive transcranial magnetic stimulation
SEA: Southeast Asia
US FDA: United States Food and Drug Administration

## References

[1] World Health Organization. Suicide in the world: global health estimates: World Health Organization; 2019. Available from: https://apps.who.int/iris/handle/10665/326948. Accessed 20 Mar 2021

[2] World Health Organization. Preventing suicide: a global imperative: World Health Organization; 2014. Available from: https://www.who.int/mental_health/suicide-prevention/world_report_2014/en/. Accessed 18 May 2021

[3] Orsolini L, Latini R, Pompili M, Serafini G, Volpe U, Vellante F (2020). Understanding the complex of suicide in depression: from research to clinics. Psychiatry Investig.

[4] Ribeiro JD, Huang X, Fox KR, Franklin JC (2018). Depression and hopelessness as risk factors for suicide ideation, attempts and death: meta-analysis of longitudinal studies. Br J Psychiatry.

[5] Osby U, Brandt L, Correia N, Ekbom A, Sparén P (2001). Excess mortality in bipolar and unipolar disorder in Sweden. Arch Gen Psychiatry.

[6] Sokero TP, Melartin TK, Rytsälä HJ, Leskelä US, Lestelä-Mielonen PS, Isometsä ET (2003). Suicidal ideation and attempts among psychiatric patients with major depressive disorder. J Clin Psychiatry.

[7] Dong M, Zeng LN, Lu L, Li XH, Ungvari GS, Ng CH (2019). Prevalence of suicide attempt in individuals with major depressive disorder: a meta-analysis of observational surveys. Psychol Med.

[8] Park S, Lee MS, Hahn SW, Si TM, Kanba S, Chong MY, Yoon CK (2016). Suicidal thoughts/acts and clinical correlates in patients with depressive disorders in Asians: results from the REAP-AD study. Acta Neuropsychiatr.

[9] Maramis MM, Pantouw JG, Lesmana CBJ. Depression screening in Surabaya Indonesia: Urgent need for better mental health care for high-risk communities and suicide prevention for men. Int J Soc Psychiatry. 2021;67(5):421–31. 10.1177/0020764020957359 32998601

[10] Chan LF, Maniam T, Shamsul AS (2011). Suicide attempts among depressed inpatients with depressive disorder in a Malaysian sample. Psychosocial and clinical risk factors. Crisis.

[11] Khan TM, Sulaiman SA, Hassali MA (2012). Factors associated with suicidal behavior among depressed patients in Penang, Malaysia. Arch Med Sci.

[12] Lim A, Lee AR, Hatim A, Tian-Mei S, Liu CY, Jeon HJ (2014). Clinical and sociodemographic correlates of suicidality in patients with major depressive disorder from six Asian countries. BMC Psychiatry.

[13] Subramaniam M, Abdin E, Seow EL, Picco L, Vaingankar JA, Chong SA (2014). Suicidal ideation, suicidal plan and suicidal attempts among those with major depressive disorder. Ann Acad Med Singap.

[14] Chen YY, Wu KC, Yousuf S, Yip PS (2012). Suicide in Asia: opportunities and challenges. Epidemiol Rev.

[15] Chu C, Van Orden KA, Ribeiro JD, Joiner TE (2017). Does the timing of suicide risk assessments influence ratings of risk severity?. Prof Psychol Res Pr.

[16] Richards JE, Whiteside U, Ludman EJ, Pabiniak C, Kirlin B, Hidalgo R, Simon G (2019). Understanding why patients may not report suicidal ideation at a health care visit prior to a suicide attempt: a qualitative study. Psychiatr Serv.

[17] Turecki G, Brent DA, Gunnell D, O'Connor RC, Oquendo MA, Pirkis J, Stanley BH (2019). Suicide and suicide risk. Nat Rev Dis Primers.

[18] American Psychiatric Association (2010). Clinical practice guideline for the treatment of patients with major depressive disorder.

[19] Lam RW, McIntosh D, Wang J, Enns MW, Kolivakis T, Michalak EE (2016). Canadian network for mood and anxiety treatments (CANMAT) 2016 clinical guidelines for the management of adults with major depressive disorder: section 1. Disease burden and principles of care. Can J Psychiatr.

[20] National Institute for Health & Clinical Excellence (2020). The NICE guideline on the treatment and management of depression in adults.

[21] Singapore Ministry of Health (2012). Depression Clinical Practice Guidelines.

[22] Malaysia Ministry of Health, Malaysian Psychiatric Association, Academy of Medicine Malaysia (2019). Clinical Practice Guidelines for the management of major depressive disorder.

[23] Lolito M, Cook E (2015). A review of suicide risk assessment instruments and approaches. Ment Health Clin.

[24] Gabriel FC, de Melo DO, Fráguas R, Leite-Santos NC, Mantovani da Silva RA, Ribeiro E (2020). Pharmacological treatment of depression: a systematic review comparing clinical practice guideline recommendations. PLoS One.

[25] Nair R, Aggarwal R, Khanna D (2011). Methods of formal consensus in classification/diagnostic criteria and guideline development. Semin Arthritis Rheum.

[26] Bennabi D, Charpeaud T, Yrondi A, Genty JB, Destouches S, Lancrenon S (2019). Clinical guidelines for the management of treatment-resistant depression: French recommendations from experts, the French Association for Biological Psychiatry and Neuropsychopharmacology and the foundation FondaMental. BMC Psychiatry.

[27] Silverman MM (2014). Suicide risk assessment and suicide risk formulation: essential components of the therapeutic risk management model. J Psychiatr Pract.

[28] Ng CWM, How CH, Ng YP (2017). Depression in primary care: assessing suicide risk. Singap Med J.

[29] Runeson B, Odeberg J, Pettersson A, Edbom T, Jildevik Adamsson I, Waern M (2017). Instruments for the assessment of suicide risk: a systematic review evaluating the certainty of the evidence. PLoS One.

[30] Wortzel HS, Homaifar B, Matarazzo B, Brenner LA (2014). Therapeutic risk management of the suicidal patient: stratifying risk in terms of severity and temporality. J Psychiatr Pract.

[31] Pisani AR, Murrie DC, Silverman MM (2016). Reformulating suicide risk formulation: from prediction to prevention. Acad Psychiatry.

[32] Wasserman D, Rihmer Z, Rujescu D, Sarchiapone M, Sokolowski M, Titelman D (2012). The European Psychiatric Association (EPA) guidance on suicide treatment and prevention. Eur Psychiatry.

[33] Milev RV, Giacobbe P, Kennedy SH, Blumberger DM, Daskalakis ZJ, Downar J (2016). Canadian network for mood and anxiety treatments (CANMAT) 2016 clinical guidelines for the management of adults with major depressive disorder: section 4. Neurostimulation treatments. Can J Psychiatr.

[34] Malhi GS, Bell E, Bassett D, Boyce P, Bryant R, Hazell P (2021). The 2020 Royal Australian and new Zealand College of Psychiatrists clinical practice guidelines for mood disorders. Aust N Z J Psychiatry.

[35] Cleare A, Pariante CM, Young AH, Anderson IM, Christmas D, Cowen PJ (2015). Evidence-based guidelines for treating depressive disorders with antidepressants: a revision of the 2008 British Association for Psychopharmacology guidelines. J Psychopharmacol.

[36] Fornaro M, Anastasia A, Valchera A, Carano A, Orsolini L, Vellante F (2019). The FDA “black box” warning on antidepressant suicide risk in young adults: more harm than benefits?. Front Psychiatry.

[37] Dold M, Kasper S (2017). Evidence-based pharmacotherapy of treatment-resistant unipolar depression. Int J Psychiatry Clin Pract.

[38] Bushnell GA, Stürmer T, Gaynes BN, Pate V, Miller M (2017). Simultaneous antidepressant and benzodiazepine new use and subsequent long-term benzodiazepine use in adults with depression, United States, 2001–2014. JAMA Psychiatry.

[39] Ogawa Y, Takeshima N, Hayasaka Y, Tajika A, Watanabe N, Streiner D, Furukawa TA (2019). Antidepressants plus benzodiazepines for adults with major depression. Cochrane Database Syst Rev.

[40] Wright BM, Eiland EH, Lorenz R (2013). Augmentation with atypical antipsychotics for depression: a review of evidence-base support from the medical literature. Pharmacotherapy.

[41] D’Anci KE, Uhl S, Giradi G, Martin C (2019). Treatments for the prevention and management of suicide: a systematic review. Ann Intern Med.

[42] Calati R, Courtet P (2016). Is psychotherapy effective for reducing suicide attempt and non-suicidal self-injury rates? Meta-analysis and meta-regression of literature data. J Psychiatr Res.

[43] Hawton K, Witt KG, Salisbury TLT, Arensman E, Gunnell D, Hazell P (2016). Psychosocial interventions following self-harm in adults: a systematic review and meta-analysis. Lancet Psychiatry.

[44] Méndez-Bustos P, Calati R, Rubio-Ramírez F, Olié E, Courtet P, Lopez-Castroman J (2019). Effectiveness of psychotherapy on suicidal risk: a systematic review of observation studies. Front Psychol.

[45] Dunlop BW (2016). Evidence-based applications of combination psychotherapy and pharmacotherapy for depression. Focus (Am Psychiatr Publ).

[46] Machado-Vieira R, Baumann J, Wheeler-Castillo C, Latov D, Henter ID, Salvadore G, Zarate CA (2010). The timing of antidepressant effects: a comparison of diverse pharmacological and somatic treatments. Pharmaceuticals (Basel).

[47] Nelson JC, Baumann P, Delucchi K, Joffe R, Katona C (2014). A systematic review and meta-analysis of lithium augmentation of tricyclic and second -generation antidepressants in major depression. J Affect Disord.

[48] Guzetta F, Tondo L, Centorrino F, Baldessarini RJ (2007). Lithium treatment reduces suicide risk in recurrent major depressive disorder. J Clin Psychiatry.

[49] Cipriani A, Hawton K, Stockton S, Geddes JR (2013). Lithium in the prevention of suicide in mood disorders: updated systematic review and meta-analysis. BMJ.

[50] Tondo L, Baldessarini RJ (2016). Suicidal behavior in mood disorders: response to pharmacological treatment. Curr Psychiatry Rep.

[51] Malhi GS, Morris G, Bell E, Hamilton A. A new paradigm for achieving a rapid antidepressant response. Drugs. 2020;80(8):75564. 10.1007/s40265-020-01303-1 32347475

[52] Davis LL, Ryan W, Adinoff B, Petty F (2000). Comprehensive review of the psychiatric uses of valproate. J Clin Psychopharmacol.

[53] Klonsky ED, May A (2010). Rethinking impulsivity in suicide. Suicide Life Threat Behav.

[54] Domany Y, Shelton RC, McCullumsmith CB (2020). Ketamine for acute suicidal ideation. An emergency department intervention: a randomized, double-blind, placebo-controlled, proof-of-concept trial. Depress Anxiety.

[55] Dadiomov D, Lee K (2019). The effects of ketamine on suicidality across various formulations and study settings. Ment Health Clin.

[56] Lascelles K, Marzano L, Brand F, Trueman H, McShane R, Hawton K (2019). Effects of ketamine treatment on suicidal ideation: a qualitative study of patients’ accounts following treatment for depression in a UK ketamine clinic. BMJ Open.

[57] Chan LF, Eu CL, Soh SY, Maniam T, Kadir ZS, Chong BT, Loo JL, Sharip S, Wong VC, Loo TH, Ng YP. Is ketamine the future clozapine for depression? A case series and literature review on maintenance ketamine in treatment-resistant depression with suicidal behavior. J Psychiatr Pract. 2018;24(4):279–291. 10.1097/PRA.0000000000000316 30427812

[58] United States Food and Drug Administration (2020). Spravato prescribing information.

[59] European Medicines Agency (2021). Spravato.

[60] Malaysia National Pharmaceutical Regulatory Agency (2021). New products approved DCA354.

[61] Singapore Health Sciences Authority. New drug approvals – Oct 2020. Available from: https://www.hsa.gov.sg/announcements/new-drug-approval/new-drug-approvals%2D%2D-oct-2020. Accessed 12 April 2021.

[62] Indonesia Badan Pengawas Obat dan Makanan RI. 2021. Available from: https://cekbpom.pom.go.id/index.php/home/produk/9uovm68qit922rsgvv6k2s3m23/all/row/10/page/1/order/4/DESC/search/5/esketamine.

[63] Fu DJ, Ionescu DF, Li X, Lane R, Lim P, Sanacora G (2020). Esketamine nasal spray for rapid reduction of major depressive disorder symptoms in patients who have active suicidal ideation with intent: double-blind, randomized study (ASPIRE I). J Clin Psychiatry.

[64] Ionescu DF, Fu DJ, Qiu X, Lane R, Lim P, Kasper S (2021). Esketamine nasal spray for rapid reduction of major depressive symptoms in patients with major depressive disorder who have active suicide ideation with intent: results of a phase 3, double-blind, randomized study (ASPIRE II). Int J Neuropsychopharmacol.

[65] Jelovac A, Kolshus E, McLoughlin DM (2013). Relapse following successful electroconvulsive therapy for major depression: a meta-analysis. Neuropsychopharmacology.

[66] Abdelnaim MA, Langguth B, Deppe M, Mohonko A, Kreuzer PM, Poeppl TB (2020). Anti-suicidal efficacy of repetitive transcranial magnetic stimulation in depressive patients: a retrospective analysis of a large sample. Front Psychiatry.

[67] Dai L, Wang P, Zhang P, Guo Q, Du H, Li F (2020). The therapeutic effect of repetitive transcranial magnetic stimulation in elderly depression patients. Medicine (Baltimore).

[68] Erlangsen A, Lind BD, Stuart EA, Qin P, Stenager E, Larsen KJ (2015). Short-terms and long-term effects of psychosocial therapy for people after deliverate self-harm: a register-based, nationwide multicentre study using propensity score matching. Lancet.

[69] Jobes DA, Wong SA, Conrad AK, Drozd JF, Neal-Walden T (2005). The collaborative assessment and management of suicidality versus treatment as usual: a retrospective study with suicidal outpatients. Suicide Life Threat Behav.

[70] Ellis TE, Rufino KA, Allen JG (2017). A controlled comparison trial of the collaborative assessment and Management of Suicidality (CAMS) in an inpatient setting: outcomes at discharge and six-month follow-up. Psychiatry Res.

[71] Hanratty D, Kilicaslan J, Wilding H, Castle D (2019). A systematic review of efficacy of collaborative assessment and Management of Suicidality (CAMS) in managing suicide risk and deliberate self-harm in adult populations. Australas Psychiatry.

[72] Tighe J, Nicholas J, Shand F, Christensen H (2018). Efficacy of acceptance and commitment therapy in reducing suicidal ideation and deliberate self-harm: systematic review. JMIR Ment Health.

[73] Vogt KS, Norman P (2019). Is mentalization-based therapy effective in treating the symptoms of borderline personality disorder? A systematic review. Psychol Psychother.

[74] Philips B, Wennberg P, Konradsson P, Franck J (2018). Mentalization-based treatment for concurrent borderline personality disorder and substance use disorder: a randomized controlled feasibility study. Eur Addict Res.

[75] Riblet NBV, Shiner B, Young-Xu Y, Watts BV (2017). Strategies to prevent death by suicide: meta-analysis of randomized controlled trials. Br J Psychiatry.

[76] Stanley B, Brown GK, Brenner LA, Galfalvy HC, Currier GW, Knox KL (2018). Comparison of the safety planning intervention with follow-up vs usual care of suicidal patients treated in the emergency department. JAMA Psychiatry.

[77] Bryan CJ, Mintz J, Clemans TA, Leeson B, Burch TS, Williams SR, Maney E, Rudd MD (2017). Effect of crisis response planning versus contracts for safety on suicide risk in US Army soldiers: a randomized clinical trial. J Affect Disord.

[78] O’Connor RC, Ferguson E, Scott F, Smyth R, McDaid D, Park AL (2017). A brief psychological intervention to reduce repetition of self-harm in patients admitted to hospital following a suicide attempt: a randomized controlled trial. Lancet Psychiatry.

[79] Gysin-Maillart A, Schwab S, Soravia L, Megert M, Michel K (2016). A novel brief therapy for patients who attempt suicide: a 24-months follow-up randomized controlled study of the attempted suicide short intervention program (ASSIP). PLoS Med.

[80] Stanley B, Brown GK (2012). Safety planning intervention: a brief intervention to mitigate suicide risk. Cognit Behv Pract.

[81] Edwards SJ, Sachmann MD (2010). No-suicide contracts, no-suicide agreements, and no-suicide assurances: a study of their nature, utilization, perceived effectiveness and potential to cause harm. Crisis.

[82] Alphs L, Fu DJ, Williamson D, Turkoz I, Jamieson C, Revicki D (2020). Suicide ideation and behavior assessment tool (SIBAT): evaluation of intra- and inter-rater reliability, validity and mapping to Columbia classification algorithm of suicide assessment. Psychiatry Res.

[83] Fonseka TM, Bhat V, Kennedy SH (2019). The utility of artificial intelligence in suicide risk prediction and the management of suicidal behaviors. Aust N Z J Psychiatry.

[84] D’Hotman D, Loh E (2020). AI enabled suicide prediction tools: a qualitative narrative review. BMJ Health Care Inform.

